# Crystal structure of diaphorin methanol monosolvate isolated from *Diaphorina citri* Kuwayama, the insect vector of citrus greening disease

**DOI:** 10.1107/S2056989018002992

**Published:** 2018-03-02

**Authors:** D. Marian Szebenyi, Irina Kriksunov, Kevin J. Howe, John S. Ramsey, David G. Hall, Michelle L. Heck, Stuart B. Krasnoff

**Affiliations:** a Cornell High Energy Synchrotron Source, Cornell University, Ithaca, NY 14853, USA; b USDA-ARS RW Holley Center for Agriculture and Health, Ithaca NY 14853, USA; c U.S. Horticultural Research Laboratory, Fort Pierce, FL 34945, USA; d Boyce Thompson Institute for Plant Research, Ithaca, NY 14853, USA; e Plant Pathology and Plant-Microbe Biology Section, Cornell University, Ithaca, NY 14853, USA

**Keywords:** crystal structure, diaphorin, pederin, Diaphorina citri, ‘*Candidatus* Profftella armatura’, hydrogen bonding

## Abstract

The title compound C_22_H_39_NO_9_·CH_3_OH [systematic name: (*S*)-*N*-((*S*)-{(2*S*,4*R*,6*R*)-6-[(*S*)-2,3-di­hydroxy­prop­yl]-4-hy­droxy-5,5-di­methyl­tetra­hydro-2*H*-pyran-2-yl}(hy­droxy)meth­yl)-2-hy­droxy-2-[(2*R*,5*R*,6*R*)-2-meth­oxy-5,6-dimeth­yl-4-methyl­ene­tetra­hydro-2*H*-pyran-2-yl]acetamide methanol monosolvate], was isolated from the Asian citrus psyllid, *Diaphorina citri* Kuwayama, and crystallizes in the space group *P*2_1_. ‘*Candidatus* Profftella armatura’ a bacterial endosymbiont of *D. citri*, biosynthesizes diaphorin, which is a hybrid polyketide–nonribosomal peptide comprising two highly substituted tetra­hydro­pyran rings joined by an *N*-acyl aminal bridge [Nakabachi *et al.* (2013 ▸). *Curr. Biol.* **23**, 1478–1484]. The crystal structure of the title compound establishes the complete relative configuration of diaphorin, which agrees at all nine chiral centers with the structure of the methanol monosolvate of the di-*p*-bromo­benzoate derivative of pederin, a biogenically related compound whose crystal structure was reported previously [Furusaki *et al.* (1968 ▸). *Tetra­hedron Lett.* **9**, 6301–6304]. Thus, the absolute configuration of diaphorin is proposed by analogy to that of pederin.

## Abstract 2

‘*Candidatus* Profftella armatura’ a bacterial endosymbiont of *D. citri*, biosynthesizes diaphorin, which is a hybrid polyketide–nonribosomal peptide comprising two highly substituted tetra­hydro­pyran rings joined by an *N*-acyl aminal bridge. The relative configurations of three out of its nine stereogenic centers, which could not be determined by NMR, were assigned based on the crystal structure.

## Chemical context   

Huanglongbing (HLB), also known as citrus greening disease, which destroys the marketability of citrus fruit and eventually kills the tree, is a major threat to world citrus production (Wang *et al.*, 2017 ▸; Bové, 2006 ▸). HLB is associated with plant infection by one of three fastidious bacterial species, ‘*Candidatus* Liberibacter asiaticus’, ‘*Candidatus* Liberibacter americanus’ or ‘*Candidatus* Liberibacter africanus’. All three bacteria are spread within a grove by psyllids – sap-sucking insects in the order Hemiptera. In North America, ‘*Ca*. L. asiaticus’ is transmitted by the invasive citrus pest, the Asian citrus psyllid, *Diaphorina citri* Kuwayama*.* A complex community of vertically transmitted endosymbiotic bacteria colonizes *D. citri*, whether or not the psyllids are infected with ‘*Ca.* L. asiaticus’ (Nakabachi *et al.*, 2013 ▸). Two of these *D. citri* endosymbionts, ‘*Candidatus* Profftella armatura’, and ‘*Candidatus* Carsonella rudii’ are localized to the bacteriome, an organ in the *D. citri* abdomen (Nakabachi *et al.*, 2013 ▸). While ‘*Ca*. C. rudii’ is the primary endosymbiont of many psyllid species, ‘*Ca*. P. armatura’ is only found in *D. citri* and has been detected in every *D. citri* population surveyed, worldwide (Nakabachi *et al.*, 2013 ▸). Approximately 15% of the ‘*Ca*. P. armatura’ genome is composed of a hybrid polyketide synthase (PKS)/nonribosomal peptide synthetase (NRPS) gene and associated tailoring genes dedicated to the biosynthesis of diaphorin. Because ‘*Ca*. P. armatura’ is unculturable, diaphorin is extracted directly from its *D. citri* host (Nakabachi *et al.*, 2013 ▸). Diaphorin is a hybrid poly­ketide–nonribosomal peptide in which two highly function­alized tetra­hydro­pyran rings are joined by an *N*-acyl aminal bridge. It is a tri-*O*-desmethyl analog of pederin, a potent cytotoxin deriving from an undetermined *Pseudomonas*-like endosymbiont of staphylinid beetles in the genus *Paederus* (Cardani *et al.*, 1967 ▸; Mosey & Floreancig, 2012 ▸; Cardani *et al.*, 1965 ▸; Furusaki *et al.*, 1968 ▸; Matsumoto *et al.*, 1968 ▸; Piel, 2002 ▸). Nakabachi *et al.* (2013 ▸) assigned the relative configuration of six of the nine stereogenic centers in diaphorin, but carbons 7, 10 and 17 remained unspecified. We pursued the crystal structure of diaphorin to complete the assignment of the relative configuration of the mol­ecule.
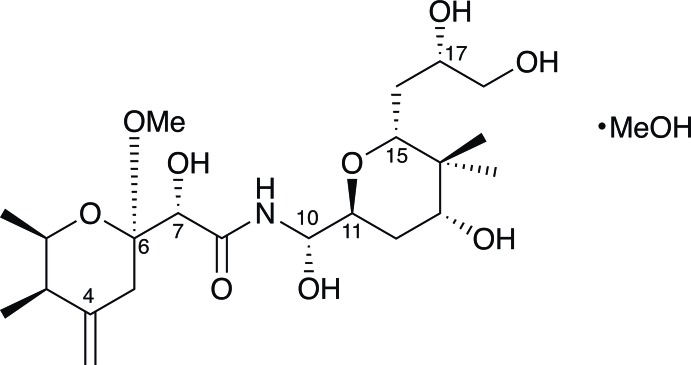


## Structural commentary   

**Figure 1:**
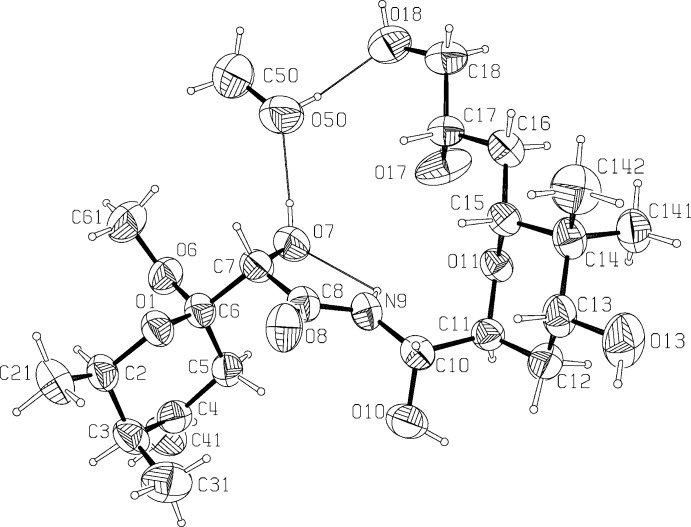
A view of the mol­ecular structure of the title compound, with the atom labeling. Displacement ellipsoids are drawn at the 50% probability level. Hydrogen bonds are shown as thin black lines (see Table 1 ▸)

The title compound crystallizes in the monoclinic *P*2_1_ space group and features an *N*-acyl aminal bridge that connects two highly substituted tetra­hydro­pyran rings adopting chair conformations (Fig. 1 ▸). Ring *A* substitutions comprise an equatorial methyl group on C2, an axial methyl group on C3, an exo­methyl­ene group on C4, and a meth­oxy group at C6. Ring *A* (O1/C2–C6) has a chair conformation with puckering parameters: amplitude *Q* = 0.541 (4) Å, θ = 173.0 (5)°, φ = 265 (4)°. Ring *B* (O11/C11–C15) substitutions comprise a hydroxyl group at C13, a geminal pair of methyl groups at C14 and a 2,3 di­hydroxy­propyl group at C15. It also has a chair conformation with puckering parameters: amplitude *Q* = 0.559 (4) Å, θ = 8.1 (4)°, φ = 258 (3)°. The mean planes of rings *A* and *B* are inclined to each other at an angle of 80.1 (2)°. For the plane including the central amide bond, (C7/O7/C8/O8/N9/C10), the r.m.s. deviation from the plane for those atoms is 0.045 Å. This planar conformation is likely influenced by a hydrogen bond in which the amide proton H9 is the donor and O7 is the acceptor with an inter­atomic distance of 2.16 Å between the participants (Fig. 1 ▸, Table 1 ▸). The chain from C13 through O18, *viz*. C13–C18/O18, is seen to be approximately planar, with an r.m.s. deviation from the plane of 0.117 Å. This conformation appears to result from crystal-packing inter­actions and probably has no biological significance. The crystal structure of the title compound assigns the three chiral centers left undetermined by Nakabachi *et al.* (2013 ▸) as 10S*, 13R*, and 17S*, and thus provides the complete relative configuration of diaphorin. The absolute configuration, as depicted in Fig. 1 ▸, was inferred by analogy to that of pederin di-*p*-bromo­benzoate (Furusaki *et al.*, 1968 ▸), which it matches at all stereogenic centers.

## Supra­molecular features   

**Figure 2:**
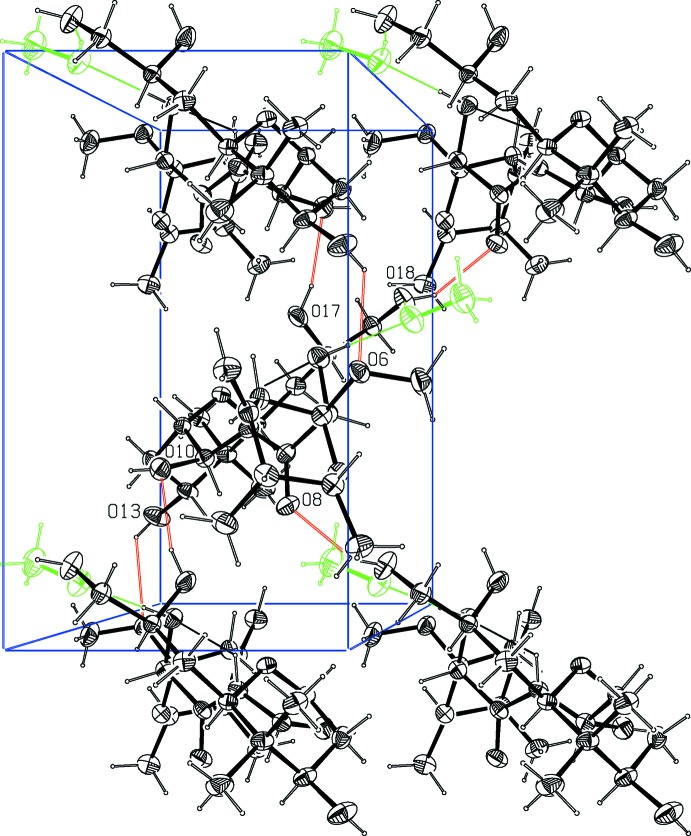
A view normal to the *ab* plane of the crystal packing of the title compound. The methanol solvent mol­ecules are shown in green and the hydrogen bonds as thin red lines (see Table 1 ▸).

The crystal structure was found to contain one methanol mol­ecule, forming two hydrogen bonds to diaphorin; the methanol OH acts as a proton donor to O18 and an acceptor of a proton from O7 (Fig. 1 ▸, Table 1 ▸). The diaphorin–methanol group forms a compact, roughly planar disk; disks are packed in a herringbone fashion as illustrated in Fig. 2 ▸. Inter­molecular contacts between symmetry-related diaphorin mol­ecules include probable hydrogen bonds between O13 (donor) and O6′ (acceptor); O17 (donor) and O10′ (acceptor); and O18 (donor) and O8′ (acceptor), as shown in Fig. 2 ▸, see also Table 1 ▸. The combination of these inter­molecular inter­actions leads to the formation of slabs lying parallel to the *ab* plane.

## Database survey   

A search of the Cambridge Structural Database (CSD, Version 5.38, update May 2017; Groom *et al.*, 2016 ▸) for related structures gave two hits. They are pederin di-*p*-bromo­benzoate methanol monosolvate (CSD refcode PEDERB; CCDC No. 1229933; Furusaki *et al.*, 1968 ▸), for which no atomic coordinates are available, and pederin di-*p*-bromo­benzoate ethanol monosolvate (CSD refcode BPEDER; CCDC No. 1114946; Corradi *et al.*, 1971 ▸). They both have the same skeleton as diaphorin, except for the addition of the two *p*-bromo­benzoate substituents. The structure of diaphorin can be matched to that of pederin by rotations about the following single bonds: C7—C8, N9—C10, C10—C11, and bonds in the C15–O18 moiety.

## Isolation and crystallization   

Diaphorin was isolated using a liquid–liquid extraction scheme with semi-preparative HPLC with modifications from the published method (Nakabachi *et al.*, 2013 ▸). A batch of *ca* 3000 *D. citri* was reared on *Citrus macrophylla* (not infected by ‘*Ca.* L. asiaticus’) at the US Horticultural Research Laboratory, Fort Pierce, FL 34945, USA. Insects were allocated to 2 ml microcentrifuge tubes, then flash frozen in liquid N_2_ and cryoground for 3.5 min at 30 Hz using 3 × 3.2 mm metal beads per tube in a ball mill apparatus (Retsch Mixer Miller MM-400). Ground insects in each tube were then extracted three times in MeOH for 45 min at 298 K. After agitation, the tubes were centrifuged for 2 min at 16,000 g, and the supernatants were pooled, filtered through two layers of Whatman #1 paper and dried *in vacuo*. The residue was taken up in 90% MeOH and partitioned against cyclo­hexane. The methano­lic phase was then fractionated by repetitive semi-preparative reversed phase HPLC using a Thermo Fluophase® column (250 × 10 mm ID, 5 µm particle), eluted at 4 ml min^−1^ with 20% MeCN, and 1 ml fractions were collected. Following detection by UV absorption at 215 nm, selected fractions were monitored for the presence of diaphorin by syringe pump infusion (5 µL min^−1^) into a Waters-Micromass ZQ single quadrupole mass spectrometer (scan range: *m*/*z* 50–1500 in 1 sec with cone and capillary voltages of 25 and 3500 V, respectively). Fractions showing the pseudomolecular ion of diaphorin (*M* + Na^+^ at *m*/*z* 484) were recombined and dried *in vacuo* to afford *ca* 4.0 mg of diaphorin. Crystals of the title compound were obtained by slow evaporation from MeOH. A single crystal measuring approximately 0.01 × 0.02 × 0.20 mm was harvested using a needle dipped in a drop of oil for adhesion (type A immersion oil, Hampton Research Corp.) and mounted in a small nylon loop (Hampton). The identity and purity of diaphorin was confirmed by comparing ^1^H NMR data acquired using the sample that afforded crystals with published data (Nakabachi *et al.*, 2013 ▸). Further confirmation was obtained by HPLC with detection by high resolution electrospray mass spectrometry (HRESIMS). Retention time (*t*_R_) and accurate mass estimates were compared with those of authentic diaphorin using a Waters Acquity UPLC system with a Waters C18 BEH column (2.1 × 50 mm; 1.7 µm), eluted at 0.3 ml min^−1^ using a gradient formed from 0.1% formic acid (A) and aceto­nitrile (B) with 0.1% formic acid (90% A 0–1 min, 14 min linear ramp to 80% A, followed by a 1 min ramp to 10% A, a 2 min hold, and a ramp back to 90% A in 1 min). Spectra were acquired on a Waters Xevo G-2 QTOF mass spectrometer operated in positive ion mode scanning the mass range from *m*/*z* 50 to 1200 in 0.1 sec with capillary and cone voltages set at 3.5 V and 25 k V, respectively. The spectrometer was calibrated in the range *m*/*z* 50–1200 using sodium formate. Spectra were calibrated in real-time using the *M* + H^+^ of co-infused leucine encephalin (*m*/*z* 556.2771) as the reference and were further processed by centering using the proprietary ‘automatic peak detection’ tool supplied with Waters MassLynx® 4.1 software.

^1^H NMR (AVIII HD 500, Bruker BioSpin, Rheinstetten Germany, 500 MHz, CD_3_OD), referenced to the center of the residual CHD_2_OD pentet at δ_H_ 3.31. δ_H_ (p.p.m.) 5.60 (*d*, *J* = 7.9 Hz, 1H, H-10), 4.80 (*t*, *J* = 2.2 Hz, 1H, H-4-CHa), 4.64 (*t*, *J* = 2.2 Hz, 1H, H-4-CHb), 4.26 (*s*, 1H, H-7), 3.882 (*m*, 2H, H-2), 3.880 (*m*, 2H, H-11), 3.76 (*qd*, *J* = 4.0, 6.3 Hz, 1H, H-17), 3.61 (*dd*, *J* = 4.4, 10.3 Hz, 1H, H-13), 3.49 (*dd*, *J* = 4.1, 11.2 Hz, 1H, H-18a), 3.40 (*m*, 2H, 15, H-18b), 3.25 (*s*, 3H, H-6-OCH_3_), 2.50 (*dt*, *J* = 2.2, 14.3 Hz, 1H, H-5 ax), 2.31 (*d*, *J* = 14.3 Hz, 1H, H-5 eq), 2.20 (*qd*, *J* = 2.5, 7.0 Hz, 1H, H-3), 2.04 (*ddd*, *J* = 3.3, 4.4, 13.5 Hz, 1H, H-12eq), 1.76 (*ddd*, *J* = 5.9, 10.3, 13.5 Hz, 1H, H-12ax), 1.67 (*t*, *J* = 6.2 Hz, 2H, H-16), 1.17 (*d*, *J* = 6.6 Hz, 3H, H-2–CH_3_), 0.99 (*d*, *J* = 7.0 Hz, 3H, H-3–CH_3_), 0.95 (*s*, 3H, H-14–CH_3_ eq), 0.88 (*s*, 3H, H-14–CH_3_ ax). HRESIMS *m*/*z* 484.2521 (calculated for C_22_H_39_NO_9_Na, 484.2517); *t*_R_ = 8.61 min.

## Refinement   

Crystal data, data collection and structure refinement details are summarized in Table 2 ▸. The hydrogen atoms were fixed geometrically (O—H = 0.84 Å, N—H = 0.86 Å, C—H = 0.98–0.10 Å) and allowed to ride on their parent atoms with *U*_iso_(H) = 1.5*U*_eq_(*C*-methyl, O-hydrox­yl) and 1.2*U*_eq_(N, C) for other H atoms.

The absolute structure of the mol­ecule in the crystal could not be determined by resonant scattering. It was assigned by analogy to that of pederin di-*p*-bromo­benzoate methanol monosolvate (Furusaki *et al.*, 1968 ▸), for which no atomic coordinates are available, and pederin di-*p*-bromo­benzoate ethanol monosolvate (Corradi *et al.*, 1971 ▸), for which the absolute configurations were determined by resonant scattering.

X-ray crystallographic data were collected at the Cornell High Energy Synchrotron Source (Ithaca, NY, 14853, USA). The synchrotron beamline available to us (CHESS F1) is normally used for macromolecular data collection. It is a fixed-wavelength line and it is not possible (due to inter­ference with equipment including the crystal-mounting robot) to move the area detector (Pilatus 6M) close enough to the sample to record data beyond 0.95 Å (in the corners; only to 1.15 Å at the edges). This explains the lack of high-resolution data, and the large s.u.’s on the cell dimensions, which may also be related to the use of the program *XDS*, which is typically used for macromolecular data reduction, for refinement of these and other experimental parameters.

## Supplementary Material

Crystal structure: contains datablock(s) I, Global. DOI: 10.1107/S2056989018002992/su5424sup1.cif

Structure factors: contains datablock(s) I. DOI: 10.1107/S2056989018002992/su5424Isup2.hkl

Supporting information file. DOI: 10.1107/S2056989018002992/su5424Isup3.cml

CCDC reference: 1824900

Additional supporting information:  crystallographic information; 3D view; checkCIF report

## Figures and Tables

**Table 1:** Hydrogen-bond geometry (Å, °)

*D*—H⋯*A*	*D*—H	H⋯*A*	*D*⋯*A*	*D*—H⋯*A*
N9—H9⋯O7	0.88	2.16	2.594 (12)	109
O7—H7*O*⋯O50	0.84	1.83	2.672 (15)	178
O50—H50*O*⋯O18	0.84	1.85	2.651 (11)	160
O13—H13*O*⋯O6^i^	0.84	2.50	2.927 (12)	113
O17—H17*O*⋯O10^ii^	0.84	2.03	2.796 (11)	152
O18—H18*O*⋯O8^iii^	0.84	2.04	2.708 (14)	136

Symmetry codes: (i) 
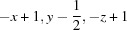
; (ii) 
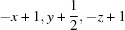
; (iii) 
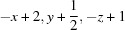
.

**Table 2:** Experimental details

Crystal data	
Chemical formula	C_22_H_39_NO_9_·CH_4_O
*M*_r_	493.58
Crystal system, space group	Monoclinic, *P*2_1_
Temperature (K)	100
*a*, *b*, *c* (Å)	7.40 (5), 12.87 (5), 13.92 (5)
β (°)	101.9 (5)
*V* (Å^3^)	1297 (11)
*Z*	2
Radiation type	Synchrotron, λ = 0.9768 Å
μ (mm^−1^)	0.10
Crystal size (mm)	0.20 × 0.02 × 0.01

Data collection	
Diffractometer	Single-axis goniometer with Dectris Pilatus 6M detector
Absorption correction	Empirical (using intensity measurements) *XDS* (Kabsch, 2010 ▸), determined correction factors as a function of position on detector surface and frame number
No. of measured, independent and observed [*I* > 2σ(*I*)] reflections	7818, 2610, 2594
*R*_int_	0.054
θ_max_ (°)	31.0
(sin θ/λ)_max_ (Å^−1^)	0.527

Refinement	
*R*[*F* ^2^ > 2σ(*F* ^2^)], *wR*(*F* ^2^), *S*	0.044, 0.110, 1.06
No. of reflections	2610
No. of parameters	309
No. of restraints	4
H-atom treatment	H-atom parameters constrained
Δρ_max_, Δρ_min_ (e Å^−3^)	0.32, −0.20

Computer programs: *ADX* (Szebenyi *et al.*, 1997 ▸), *HKL-2000* (Otwinowski & Minor, 1997 ▸), *XDS* (Kabsch, 2010 ▸), *SnB* (Weeks & Miller, 1999*a* ▸,*b* ▸), *COOT* (Emsley *et al.*, 2010 ▸), *ORTEPIII* (Burnett & Johnson, 1996 ▸), *SHELXL2016* (Sheldrick, 2015 ▸), *PLATON* (Spek, 2009 ▸) and *publCIF* (Westrip, 2010 ▸).

## supplementary crystallographic information

### Crystal data

**Table:**

C_22_H_39_NO_9_·CH_4_O	*F*(000) = 536
*M**_r_* = 493.58	*D*_x_ = 1.263 Mg m^−^^3^
Monoclinic, *P*2_1_	Synchrotron radiation, λ = 0.9768 Å
*a* = 7.40 (5) Å	Cell parameters from 7493 reflections
*b* = 12.87 (5) Å	µ = 0.10 mm^−^^1^
*c* = 13.92 (5) Å	*T* = 100 K
β = 101.9 (5)°	Needle, colorless
*V* = 1297 (11) Å^3^	0.20 × 0.02 × 0.01 mm
*Z* = 2	

### Data collection

**Table:**

Single-axis goniometer diffractometer	2610 independent reflections
Radiation source: synchrotron, CHESS F1	2594 reflections with *I* > 2σ(*I*)
Si 111 monochromator	*R*_int_ = 0.054
Detector resolution: 5.8 pixels mm^-1^	θ_max_ = 31.0°, θ_min_ = 2.1°
rotation scans	*h* = −7→7
Absorption correction: empirical (using intensity measurements) *XDS* (Kabsch, 2010), determined correction factors as a function of position on detector surface and frame number	*k* = −11→11
	*l* = −13→13
7818 measured reflections	

### Refinement

**Table:**

Refinement on *F*^2^	Hydrogen site location: inferred from neighbouring sites
Least-squares matrix: full	H-atom parameters constrained
*R*[*F*^2^ > 2σ(*F*^2^)] = 0.044	*w* = 1/[σ^2^(*F*_o_^2^) + (0.0824*P*)^2^ + 0.1123*P*] where *P* = (*F*_o_^2^ + 2*F*_c_^2^)/3
*wR*(*F*^2^) = 0.110	(Δ/σ)_max_ = 0.003
*S* = 1.06	Δρ_max_ = 0.32 e Å^−^^3^
2610 reflections	Δρ_min_ = −0.20 e Å^−^^3^
309 parameters	Extinction correction: (SHELXL2016; Sheldrick, 2015), Fc^*^=kFc[1+0.001xFc^2^λ^3^/sin(2θ)]^-1/4^
4 restraints	Extinction coefficient: 0.40 (2)

### Special details

**Table:**

Geometry. All esds (except the esd in the dihedral angle between two l.s. planes) are estimated using the full covariance matrix. The cell esds are taken into account individually in the estimation of esds in distances, angles and torsion angles; correlations between esds in cell parameters are only used when they are defined by crystal symmetry. An approximate (isotropic) treatment of cell esds is used for estimating esds involving l.s. planes.

### Fractional atomic coordinates and isotropic or equivalent isotropic displacement parameters (Å^2^)

**Table:**

	*x*	*y*	*z*	*U*_iso_*/*U*_eq_	
O1	0.9096 (3)	0.2920 (2)	0.80732 (18)	0.0500 (8)	
C2	0.9272 (6)	0.2740 (4)	0.9119 (3)	0.0535 (11)	
H2	1.016017	0.326420	0.947691	0.064*	
C21	1.0113 (7)	0.1696 (4)	0.9330 (3)	0.0699 (14)	
H21A	1.025710	0.154301	1.003109	0.105*	
H21B	0.931223	0.117144	0.894720	0.105*	
H21C	1.132612	0.168310	0.915028	0.105*	
C3	0.7419 (6)	0.2909 (4)	0.9412 (3)	0.0573 (12)	
H3	0.763070	0.286355	1.014381	0.069*	
C31	0.5998 (8)	0.2084 (4)	0.8990 (4)	0.0794 (15)	
H31A	0.648769	0.139379	0.919953	0.119*	
H31B	0.485812	0.220347	0.922838	0.119*	
H31C	0.573618	0.212280	0.827217	0.119*	
C4	0.6769 (5)	0.3987 (3)	0.9124 (3)	0.0526 (12)	
C41	0.6378 (7)	0.4687 (4)	0.9736 (3)	0.0729 (14)	
H41A	0.650173	0.452128	1.041137	0.087*	
H41B	0.597033	0.535848	0.950379	0.087*	
C5	0.6684 (5)	0.4217 (4)	0.8052 (3)	0.0519 (11)	
H5A	0.643771	0.496615	0.792631	0.062*	
H5B	0.565825	0.381975	0.764628	0.062*	
C6	0.8492 (5)	0.3925 (3)	0.7761 (3)	0.0486 (11)	
C61	1.1667 (6)	0.4515 (5)	0.8158 (4)	0.0846 (16)	
H61A	1.242862	0.508656	0.848400	0.127*	
H61B	1.208582	0.385864	0.848678	0.127*	
H61C	1.177943	0.447788	0.746930	0.127*	
O6	0.9777 (4)	0.4692 (2)	0.8205 (2)	0.0605 (9)	
C7	0.8314 (5)	0.3961 (4)	0.6628 (3)	0.0507 (11)	
H7	0.951763	0.375121	0.646523	0.061*	
O7	0.7924 (4)	0.4996 (3)	0.6331 (2)	0.0619 (9)	
H7O	0.877526	0.522651	0.607387	0.093*	
C8	0.6803 (5)	0.3240 (4)	0.6102 (3)	0.0464 (11)	
O8	0.6892 (4)	0.2290 (3)	0.6211 (2)	0.0632 (9)	
N9	0.5382 (4)	0.3699 (3)	0.5516 (2)	0.0497 (9)	
H9	0.543516	0.437329	0.541738	0.060*	
C10	0.3757 (5)	0.3139 (4)	0.5035 (3)	0.0480 (10)	
H10	0.413987	0.246240	0.478500	0.058*	
O10	0.2581 (4)	0.2944 (3)	0.5687 (2)	0.0664 (9)	
H10O	0.148429	0.291116	0.537269	0.100*	
C11	0.2635 (5)	0.3755 (3)	0.4177 (3)	0.0453 (10)	
H11	0.186863	0.425783	0.446886	0.054*	
O11	0.3736 (3)	0.43630 (19)	0.36578 (17)	0.0441 (7)	
C12	0.1296 (6)	0.3068 (4)	0.3494 (3)	0.0586 (12)	
H12A	0.071687	0.257717	0.388794	0.070*	
H12B	0.030483	0.350459	0.310811	0.070*	
C13	0.2208 (6)	0.2458 (3)	0.2797 (3)	0.0557 (12)	
H13	0.305741	0.193298	0.318282	0.067*	
O13	0.0833 (5)	0.1925 (3)	0.2103 (3)	0.0866 (12)	
H13O	0.024701	0.151919	0.239959	0.130*	
C14	0.3347 (6)	0.3177 (4)	0.2252 (3)	0.0523 (11)	
C141	0.2083 (6)	0.3926 (4)	0.1573 (3)	0.0640 (12)	
H81A	0.141651	0.436190	0.196215	0.096*	
H81B	0.282632	0.436814	0.123108	0.096*	
H81C	0.119616	0.352949	0.109176	0.096*	
C142	0.4403 (8)	0.2489 (4)	0.1661 (4)	0.0786 (15)	
H82A	0.520714	0.201311	0.210493	0.118*	
H82B	0.352426	0.208692	0.117926	0.118*	
H82C	0.515442	0.292557	0.131858	0.118*	
C15	0.4700 (5)	0.3774 (3)	0.3038 (3)	0.0429 (9)	
H15	0.552106	0.325972	0.345588	0.052*	
C16	0.5904 (6)	0.4549 (4)	0.2642 (3)	0.0551 (11)	
H16A	0.660085	0.417211	0.221483	0.066*	
H16B	0.508935	0.505438	0.222398	0.066*	
C17	0.7263 (5)	0.5146 (3)	0.3396 (3)	0.0472 (10)	
H17	0.795982	0.464083	0.388030	0.057*	
O17	0.6405 (4)	0.5872 (3)	0.3908 (3)	0.0862 (12)	
H17O	0.683199	0.646702	0.384383	0.129*	
C18	0.8628 (6)	0.5731 (4)	0.2932 (3)	0.0637 (13)	
H18A	0.923433	0.524690	0.254403	0.076*	
H18B	0.797692	0.627021	0.248268	0.076*	
O18	0.9944 (5)	0.6193 (3)	0.3660 (3)	0.0858 (12)	
H18O	1.090157	0.631327	0.344277	0.129*	
C50	1.2409 (8)	0.6109 (6)	0.5972 (4)	0.0977 (19)	
H50A	1.231228	0.684217	0.614718	0.147*	
H50B	1.322178	0.604524	0.550159	0.147*	
H50C	1.292065	0.570815	0.656395	0.147*	
O50	1.0684 (5)	0.5734 (3)	0.5557 (3)	0.0862 (11)	
H50O	1.032480	0.600368	0.500097	0.103*	

### Atomic displacement parameters (Å^2^)

**Table:**

	*U*^11^	*U*^22^	*U*^33^	*U*^12^	*U*^13^	*U*^23^
O1	0.0570 (16)	0.055 (2)	0.0380 (15)	0.0062 (13)	0.0094 (11)	0.0020 (12)
C2	0.061 (2)	0.059 (3)	0.037 (2)	−0.001 (2)	0.0026 (17)	0.0020 (18)
C21	0.086 (3)	0.068 (4)	0.054 (3)	0.010 (2)	0.012 (2)	0.012 (2)
C3	0.073 (3)	0.061 (3)	0.039 (2)	−0.008 (2)	0.0138 (18)	0.000 (2)
C31	0.086 (3)	0.064 (4)	0.093 (3)	−0.019 (3)	0.028 (3)	−0.007 (3)
C4	0.054 (2)	0.057 (3)	0.049 (2)	−0.006 (2)	0.0142 (19)	−0.007 (2)
C41	0.097 (3)	0.071 (3)	0.056 (3)	0.003 (3)	0.026 (2)	−0.009 (2)
C5	0.051 (2)	0.059 (3)	0.044 (2)	0.004 (2)	0.0073 (18)	−0.0005 (19)
C6	0.050 (2)	0.055 (3)	0.039 (2)	0.000 (2)	0.0058 (17)	−0.001 (2)
C61	0.050 (3)	0.103 (4)	0.095 (4)	−0.018 (3)	0.001 (2)	0.009 (3)
O6	0.0532 (17)	0.063 (2)	0.0605 (17)	−0.0105 (14)	0.0004 (13)	−0.0011 (14)
C7	0.053 (2)	0.054 (3)	0.047 (2)	0.0050 (19)	0.0138 (18)	0.007 (2)
O7	0.0651 (17)	0.063 (2)	0.0582 (17)	−0.0057 (15)	0.0128 (14)	0.0127 (15)
C8	0.052 (2)	0.050 (3)	0.038 (2)	0.010 (2)	0.0116 (19)	−0.0045 (19)
O8	0.068 (2)	0.053 (3)	0.0614 (19)	0.0169 (15)	−0.0022 (15)	−0.0052 (15)
N9	0.056 (2)	0.047 (2)	0.0433 (18)	0.0063 (16)	0.0037 (17)	0.0048 (16)
C10	0.051 (2)	0.049 (2)	0.045 (2)	0.0039 (19)	0.0124 (18)	0.0049 (19)
O10	0.0692 (18)	0.074 (2)	0.0617 (18)	0.0026 (16)	0.0263 (15)	0.0147 (15)
C11	0.046 (2)	0.045 (2)	0.046 (2)	0.0022 (18)	0.0104 (17)	0.0072 (19)
O11	0.0536 (14)	0.0383 (16)	0.0401 (14)	−0.0021 (12)	0.0093 (12)	0.0011 (11)
C12	0.048 (2)	0.063 (3)	0.061 (3)	−0.004 (2)	0.0028 (19)	0.013 (2)
C13	0.063 (2)	0.046 (3)	0.051 (2)	−0.012 (2)	−0.006 (2)	0.0020 (19)
O13	0.103 (3)	0.063 (2)	0.079 (2)	−0.031 (2)	−0.0153 (19)	−0.0031 (17)
C14	0.066 (2)	0.045 (2)	0.043 (2)	−0.002 (2)	0.0035 (19)	−0.0041 (19)
C141	0.074 (3)	0.060 (3)	0.051 (2)	−0.008 (2)	−0.003 (2)	0.003 (2)
C142	0.100 (4)	0.063 (4)	0.074 (3)	−0.008 (3)	0.020 (3)	−0.025 (3)
C15	0.051 (2)	0.035 (2)	0.042 (2)	0.0022 (18)	0.0093 (17)	0.0024 (18)
C16	0.066 (2)	0.055 (3)	0.046 (2)	−0.003 (2)	0.0125 (19)	0.003 (2)
C17	0.050 (2)	0.043 (3)	0.051 (2)	−0.0006 (18)	0.0158 (18)	0.0007 (18)
O17	0.070 (2)	0.069 (2)	0.132 (3)	−0.0264 (18)	0.051 (2)	−0.048 (2)
C18	0.061 (3)	0.063 (3)	0.072 (3)	−0.008 (2)	0.026 (2)	0.004 (2)
O18	0.0644 (19)	0.103 (3)	0.090 (3)	−0.033 (2)	0.0148 (18)	0.007 (2)
C50	0.080 (4)	0.127 (6)	0.081 (4)	−0.021 (4)	0.003 (3)	0.000 (3)
O50	0.084 (2)	0.098 (3)	0.079 (2)	−0.023 (2)	0.0224 (19)	0.006 (2)

### Geometric parameters (Å, º)

**Table:**

O1—C6	1.407 (7)	C11—O11	1.429 (7)
O1—C2	1.454 (7)	C11—C12	1.511 (9)
C2—C21	1.485 (9)	C11—H11	1.0000
C2—C3	1.525 (11)	O11—C15	1.443 (7)
C2—H2	1.0000	C12—C13	1.510 (9)
C21—H21A	0.9800	C12—H12A	0.9900
C21—H21B	0.9800	C12—H12B	0.9900
C21—H21C	0.9800	C13—O13	1.427 (9)
C3—C4	1.496 (9)	C13—C14	1.552 (8)
C3—C31	1.525 (9)	C13—H13	1.0000
C3—H3	1.0000	O13—H13O	0.8400
C31—H31A	0.9800	C14—C141	1.527 (9)
C31—H31B	0.9800	C14—C142	1.528 (9)
C31—H31C	0.9800	C14—C15	1.529 (9)
C4—C41	1.312 (7)	C141—H81A	0.9800
C4—C5	1.510 (8)	C141—H81B	0.9800
C41—H41A	0.9500	C141—H81C	0.9800
C41—H41B	0.9500	C142—H82A	0.9800
C5—C6	1.523 (11)	C142—H82B	0.9800
C5—H5A	0.9900	C142—H82C	0.9800
C5—H5B	0.9900	C15—C16	1.515 (8)
C6—O6	1.421 (8)	C15—H15	1.0000
C6—C7	1.557 (8)	C16—C17	1.507 (9)
C61—O6	1.431 (11)	C16—H16A	0.9900
C61—H61A	0.9800	C16—H16B	0.9900
C61—H61B	0.9800	C17—O17	1.403 (7)
C61—H61C	0.9800	C17—C18	1.508 (9)
C7—O7	1.406 (8)	C17—H17	1.0000
C7—C8	1.520 (9)	O17—H17O	0.8400
C7—H7	1.0000	C18—O18	1.387 (10)
O7—H7O	0.8400	C18—H18A	0.9900
C8—O8	1.231 (7)	C18—H18B	0.9900
C8—N9	1.330 (9)	O18—H18O	0.8400
N9—C10	1.444 (9)	C50—O50	1.376 (11)
N9—H9	0.8800	C50—H50A	0.9800
C10—O10	1.403 (9)	C50—H50B	0.9800
C10—C11	1.528 (9)	C50—H50C	0.9800
C10—H10	1.0000	O50—H50O	0.8400
O10—H10O	0.8400		
			
C6—O1—C2	114.4 (3)	O11—C11—C10	113.9 (5)
O1—C2—C21	106.8 (4)	C12—C11—C10	111.7 (5)
O1—C2—C3	110.2 (5)	O11—C11—H11	106.3
C21—C2—C3	116.1 (4)	C12—C11—H11	106.3
O1—C2—H2	107.8	C10—C11—H11	106.3
C21—C2—H2	107.8	C11—O11—C15	114.7 (4)
C3—C2—H2	107.8	C13—C12—C11	112.7 (5)
C2—C21—H21A	109.5	C13—C12—H12A	109.0
C2—C21—H21B	109.5	C11—C12—H12A	109.0
H21A—C21—H21B	109.5	C13—C12—H12B	109.0
C2—C21—H21C	109.5	C11—C12—H12B	109.0
H21A—C21—H21C	109.5	H12A—C12—H12B	107.8
H21B—C21—H21C	109.5	O13—C13—C12	109.5 (5)
C4—C3—C2	108.5 (4)	O13—C13—C14	109.8 (4)
C4—C3—C31	112.6 (5)	C12—C13—C14	111.3 (5)
C2—C3—C31	112.6 (5)	O13—C13—H13	108.7
C4—C3—H3	107.6	C12—C13—H13	108.7
C2—C3—H3	107.6	C14—C13—H13	108.7
C31—C3—H3	107.6	C13—O13—H13O	109.5
C3—C31—H31A	109.5	C141—C14—C142	110.4 (5)
C3—C31—H31B	109.5	C141—C14—C15	110.7 (5)
H31A—C31—H31B	109.5	C142—C14—C15	110.1 (5)
C3—C31—H31C	109.5	C141—C14—C13	110.7 (5)
H31A—C31—H31C	109.5	C142—C14—C13	107.9 (5)
H31B—C31—H31C	109.5	C15—C14—C13	106.9 (4)
C41—C4—C3	124.3 (5)	C14—C141—H81A	109.5
C41—C4—C5	122.5 (5)	C14—C141—H81B	109.5
C3—C4—C5	113.1 (4)	H81A—C141—H81B	109.5
C4—C41—H41A	120.0	C14—C141—H81C	109.5
C4—C41—H41B	120.0	H81A—C141—H81C	109.5
H41A—C41—H41B	120.0	H81B—C141—H81C	109.5
C4—C5—C6	110.8 (5)	C14—C142—H82A	109.5
C4—C5—H5A	109.5	C14—C142—H82B	109.5
C6—C5—H5A	109.5	H82A—C142—H82B	109.5
C4—C5—H5B	109.5	C14—C142—H82C	109.5
C6—C5—H5B	109.5	H82A—C142—H82C	109.5
H5A—C5—H5B	108.1	H82B—C142—H82C	109.5
O1—C6—O6	111.2 (5)	O11—C15—C16	105.8 (5)
O1—C6—C5	112.7 (4)	O11—C15—C14	111.1 (5)
O6—C6—C5	105.0 (5)	C16—C15—C14	114.7 (4)
O1—C6—C7	107.2 (4)	O11—C15—H15	108.4
O6—C6—C7	109.3 (5)	C16—C15—H15	108.4
C5—C6—C7	111.5 (5)	C14—C15—H15	108.4
O6—C61—H61A	109.5	C17—C16—C15	116.1 (4)
O6—C61—H61B	109.5	C17—C16—H16A	108.3
H61A—C61—H61B	109.5	C15—C16—H16A	108.3
O6—C61—H61C	109.5	C17—C16—H16B	108.3
H61A—C61—H61C	109.5	C15—C16—H16B	108.3
H61B—C61—H61C	109.5	H16A—C16—H16B	107.4
C6—O6—C61	116.2 (5)	O17—C17—C16	112.7 (5)
O7—C7—C8	110.5 (5)	O17—C17—C18	107.4 (5)
O7—C7—C6	107.2 (4)	C16—C17—C18	111.4 (5)
C8—C7—C6	111.7 (5)	O17—C17—H17	108.4
O7—C7—H7	109.1	C16—C17—H17	108.4
C8—C7—H7	109.1	C18—C17—H17	108.4
C6—C7—H7	109.1	C17—O17—H17O	109.5
C7—O7—H7O	109.5	O18—C18—C17	109.3 (5)
O8—C8—N9	122.2 (4)	O18—C18—H18A	109.8
O8—C8—C7	122.1 (5)	C17—C18—H18A	109.8
N9—C8—C7	115.7 (6)	O18—C18—H18B	109.8
C8—N9—C10	122.7 (5)	C17—C18—H18B	109.8
C8—N9—H9	118.6	H18A—C18—H18B	108.3
C10—N9—H9	118.6	C18—O18—H18O	109.5
O10—C10—N9	110.9 (5)	O50—C50—H50A	109.5
O10—C10—C11	106.8 (5)	O50—C50—H50B	109.5
N9—C10—C11	111.8 (5)	H50A—C50—H50B	109.5
O10—C10—H10	109.1	O50—C50—H50C	109.5
N9—C10—H10	109.1	H50A—C50—H50C	109.5
C11—C10—H10	109.1	H50B—C50—H50C	109.5
C10—O10—H10O	109.5	C50—O50—H50O	109.5
O11—C11—C12	111.7 (4)		
			
C6—O1—C2—C21	−173.8 (3)	C8—N9—C10—O10	78.8 (5)
C6—O1—C2—C3	59.3 (4)	C8—N9—C10—C11	−162.2 (3)
O1—C2—C3—C4	−57.1 (4)	O10—C10—C11—O11	156.4 (4)
C21—C2—C3—C4	−178.6 (4)	N9—C10—C11—O11	35.0 (5)
O1—C2—C3—C31	68.3 (6)	O10—C10—C11—C12	−75.9 (5)
C21—C2—C3—C31	−53.3 (6)	N9—C10—C11—C12	162.7 (3)
C2—C3—C4—C41	−122.7 (6)	C12—C11—O11—C15	−53.8 (5)
C31—C3—C4—C41	111.9 (6)	C10—C11—O11—C15	73.9 (5)
C2—C3—C4—C5	54.6 (6)	O11—C11—C12—C13	49.2 (5)
C31—C3—C4—C5	−70.7 (6)	C10—C11—C12—C13	−79.7 (5)
C41—C4—C5—C6	127.0 (5)	C11—C12—C13—O13	−173.2 (3)
C3—C4—C5—C6	−50.4 (5)	C11—C12—C13—C14	−51.6 (5)
C2—O1—C6—O6	62.7 (6)	O13—C13—C14—C141	55.7 (6)
C2—O1—C6—C5	−54.9 (5)	C12—C13—C14—C141	−65.8 (5)
C2—O1—C6—C7	−177.9 (3)	O13—C13—C14—C142	−65.2 (5)
C4—C5—C6—O1	48.9 (5)	C12—C13—C14—C142	173.3 (4)
C4—C5—C6—O6	−72.3 (5)	O13—C13—C14—C15	176.3 (3)
C4—C5—C6—C7	169.5 (4)	C12—C13—C14—C15	54.9 (5)
O1—C6—O6—C61	49.5 (5)	C11—O11—C15—C16	−174.7 (3)
C5—C6—O6—C61	171.7 (4)	C11—O11—C15—C14	60.2 (4)
C7—C6—O6—C61	−68.6 (6)	C141—C14—C15—O11	62.4 (4)
O1—C6—C7—O7	−173.2 (3)	C142—C14—C15—O11	−175.2 (3)
O6—C6—C7—O7	−52.5 (5)	C13—C14—C15—O11	−58.3 (5)
C5—C6—C7—O7	63.1 (4)	C141—C14—C15—C16	−57.5 (5)
O1—C6—C7—C8	65.6 (5)	C142—C14—C15—C16	64.9 (6)
O6—C6—C7—C8	−173.7 (3)	C13—C14—C15—C16	−178.2 (3)
C5—C6—C7—C8	−58.1 (6)	O11—C15—C16—C17	57.6 (5)
O7—C7—C8—O8	176.5 (3)	C14—C15—C16—C17	−179.6 (4)
C6—C7—C8—O8	−64.2 (5)	C15—C16—C17—O17	−70.4 (6)
O7—C7—C8—N9	−3.3 (4)	C15—C16—C17—C18	168.8 (4)
C6—C7—C8—N9	115.9 (5)	O17—C17—C18—O18	61.3 (5)
O8—C8—N9—C10	6.0 (5)	C16—C17—C18—O18	−174.8 (4)
C7—C8—N9—C10	−174.1 (3)		

### Hydrogen-bond geometry (Å, º)

**Table:**

*D*—H···*A*	*D*—H	H···*A*	*D*···*A*	*D*—H···*A*
N9—H9···O7	0.88	2.16	2.594 (12)	109
O7—H7*O*···O50	0.84	1.83	2.672 (15)	178
O50—H50*O*···O18	0.84	1.85	2.651 (11)	160
O13—H13*O*···O6^i^	0.84	2.50	2.927 (12)	113
O17—H17*O*···O10^ii^	0.84	2.03	2.796 (11)	152
O18—H18*O*···O8^iii^	0.84	2.04	2.708 (14)	136

Symmetry codes: (i) −*x*+1, *y*−1/2, −*z*+1; (ii) −*x*+1, *y*+1/2, −*z*+1; (iii) −*x*+2, *y*+1/2, −*z*+1.
